# Supplementary figures and images for: A lipoprotein allosterically activates the CwlD amidase during Clostridioides difficile spore formation
Source: PLoS Genet. 2021 Sep 27;17(9):e1009791. doi: 10.1371/journal.pgen.1009791 (PMC8496864; doi:10.1371/journal.pgen.1009791)

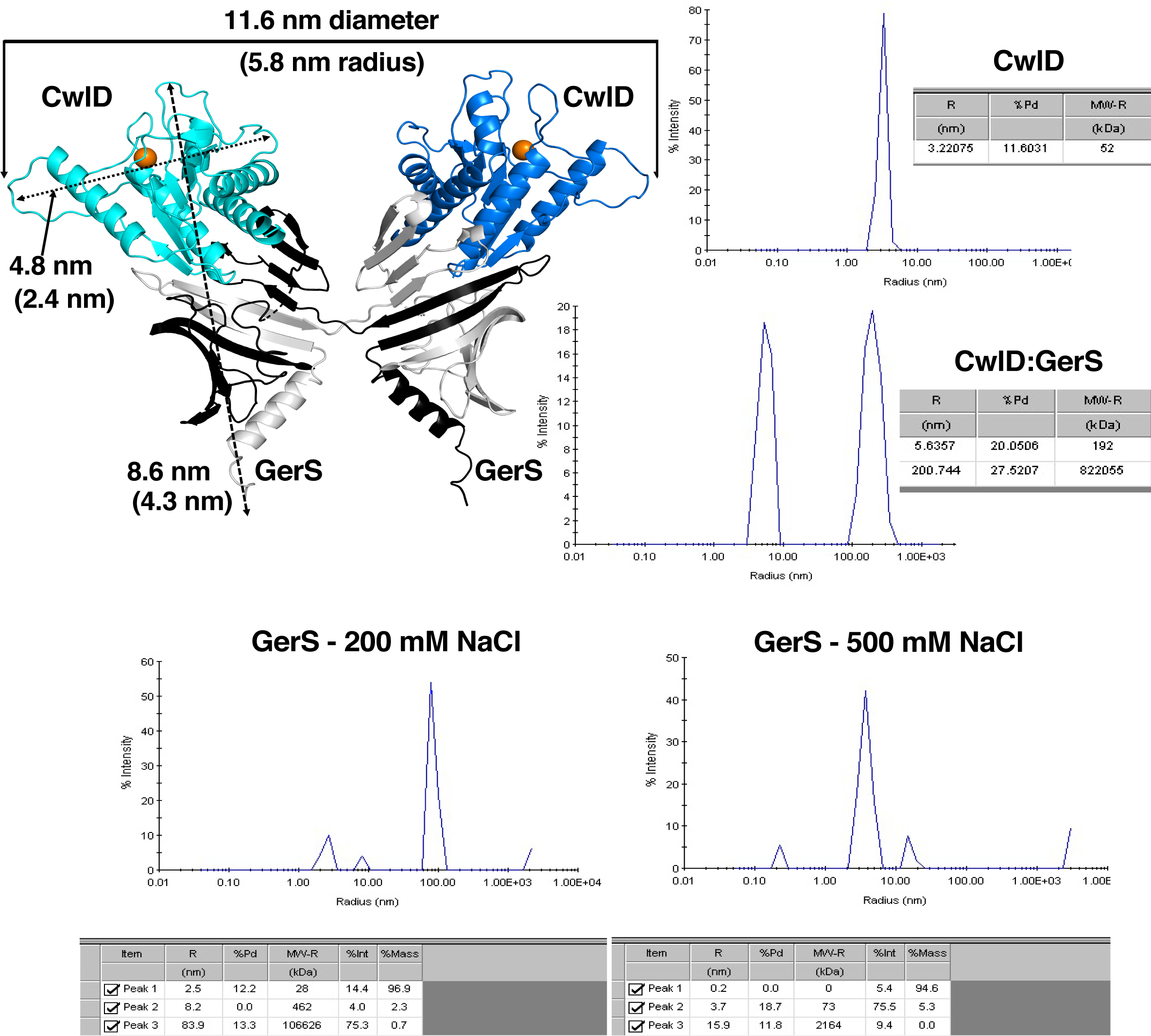

Supplement: S1 Fig — The left panel shows the two CwlD / two GerS complex within the crystal with dimensions along each axis shown in nm. Dynamic light scattering results are shown for purified CwlD, CwlD:GerS complex, and GerS. The radius of the CwlD:GerS complex in solution of 5.6 nm agrees well with 5.8 nm as measured in the crystal. The 200 nm peak in the complex sample is excess GerS not in the complex as GerS in the absence of CwlD behaves multimodally with some aggregation. Purified GerS shows multiple peaks on SEC with the expected monomer peak redistributing back into multiple peaks upon storage. At low protein concentration (1 mg/mL) and high salt (500 mM NaCl), the DLS radius of 3.7 nm (diameter of 7.4 nm) is in agreement with the largest measurement of 7.8 nm for the GerS dimer structure when excluding the two CwlD molecules. (TIF) [file pgen.1009791.s001.tif]

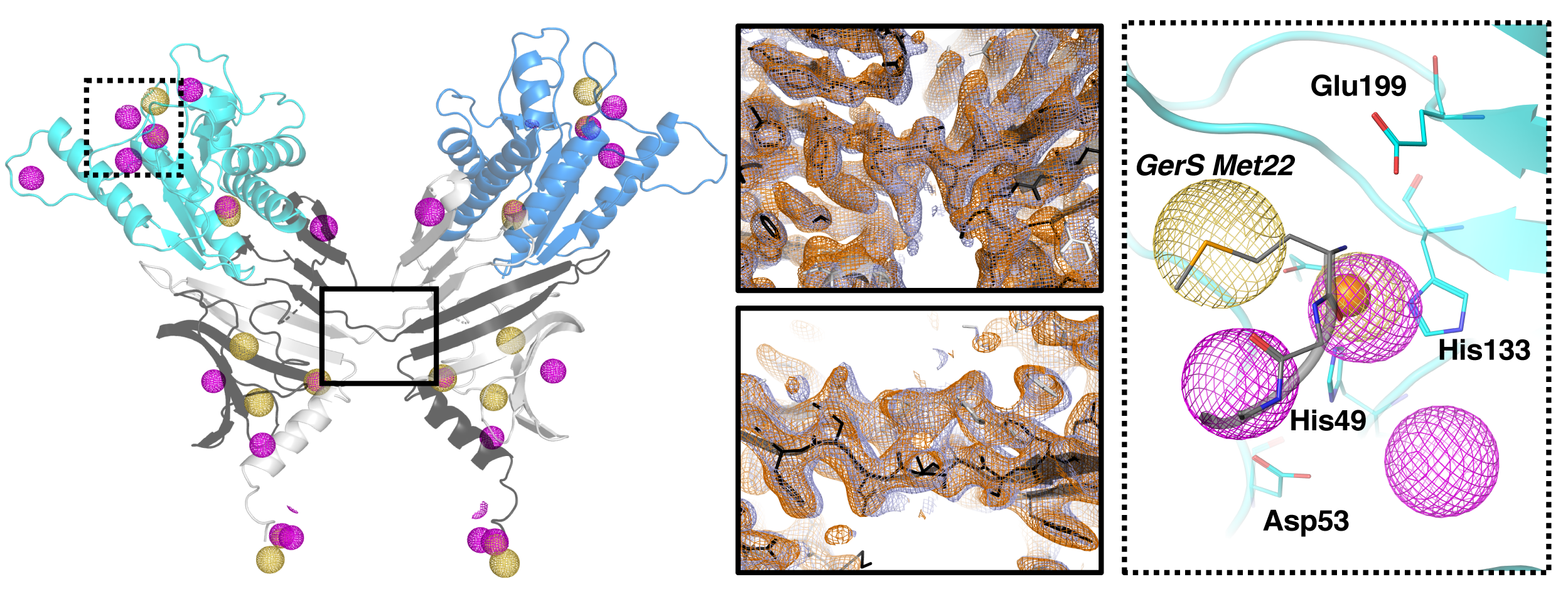

Supplement: S2 Fig — Left shows the two CwlD (Blue tones) / 2 GerS (grey tones) complex. The yellow mesh shows the anomalous signal at 3 sigma for the selenomethionine derivative and the maroon the sodium iodide derivative from Autosharp (Global Phasing Limited). Middle panels show Se-SAD phases in blue and Se/NaI with native MIR phases in orange contoured at 1 sigma and the beta strand crossover between the two GerS protomers (solid box) with views 90o apart. Right panel (dotted box) shows the anomalous peaks near the zinc binding site of CwlD. (TIF) [file pgen.1009791.s002.tif]

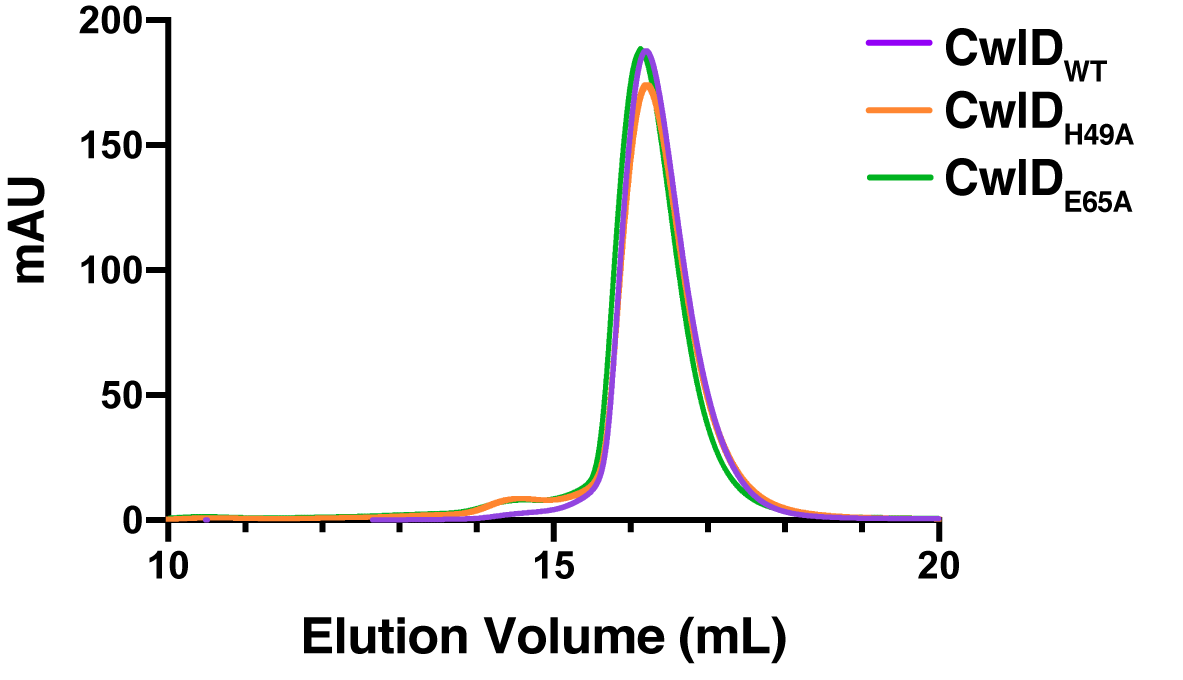

Supplement: S3 Fig — Purified His-tagged CwlDWT, CwlDH49A, and CwlDE65A were analyzed by size exclusion chromatography. mAU corresponds to the UV absorbance measurements (A280) during the protein elution. (TIF) [file pgen.1009791.s003.tif]

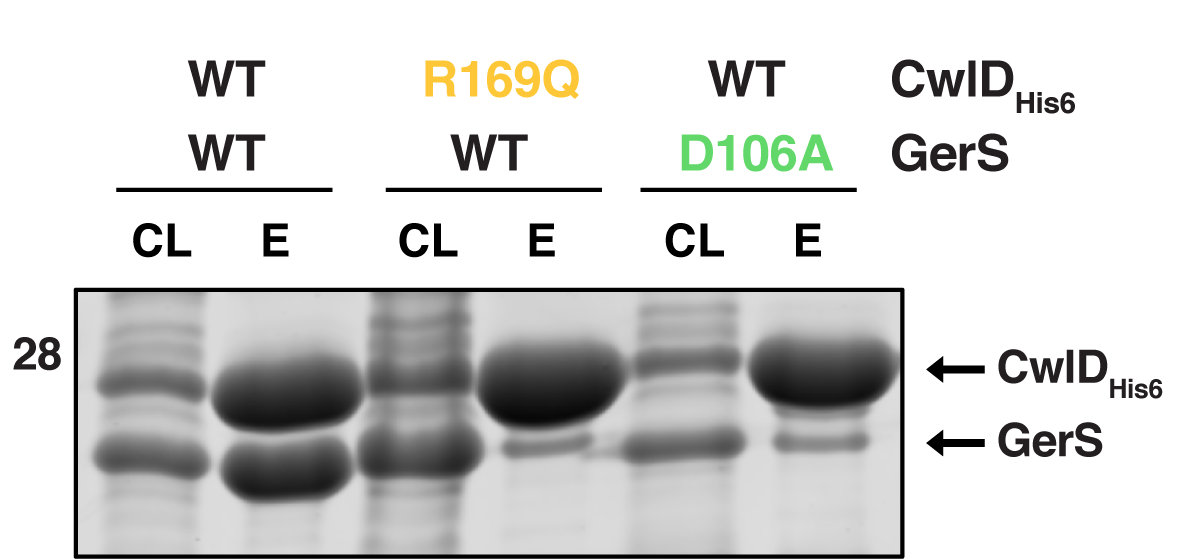

Supplement: S4 Fig — The indicated proteins were produced in E. coli and purified using Ni2+- affinity resin. Cleared lysate (CL) and eluate (E) fractions were analyzed using Coomassie staining. (TIF) [file pgen.1009791.s004.tif]

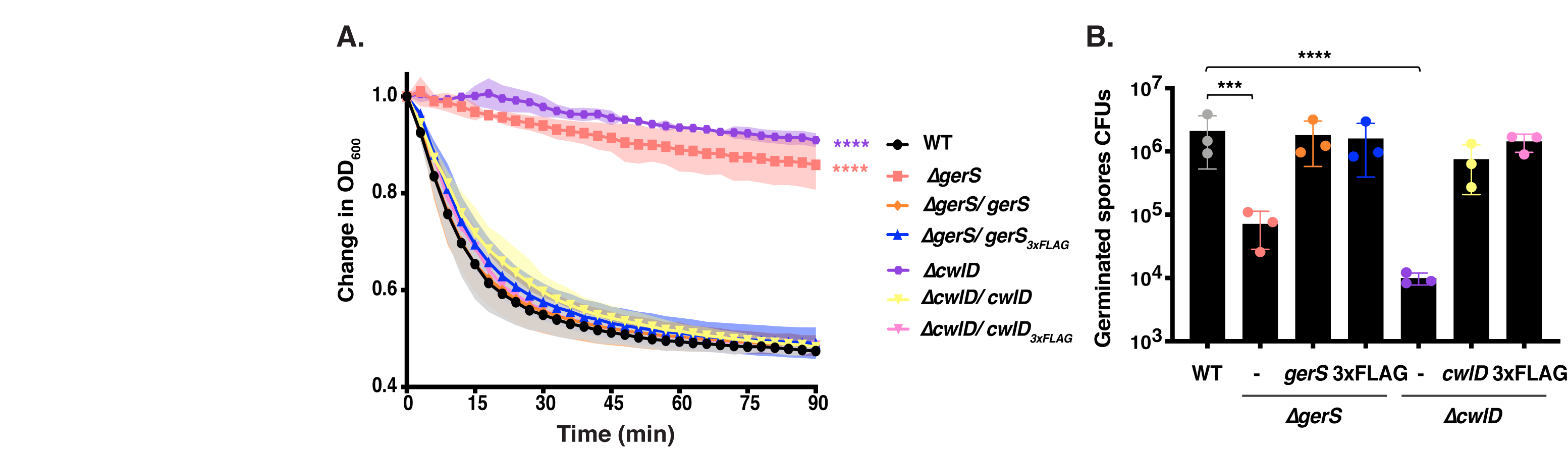

Supplement: S5 Fig — (A) Change in the OD600 in response to germinant of ΔgerS and ΔcwlD spores complemented with gerSFLAG and cwlDFLAG constructs, respectively, relative to spores complementated with native gerS or cwlD, respectively. Purified spores were resuspended in BHIS, and germination was induced by adding taurocholate (1% final concentration). The ratio of the OD600 of each strain at a given time point relative to the OD600 at time zero is plotted. The mean of three assays from 3 independent spore preparations are shown. Shading represents the standard deviation as the area between error bars for each time point measured. Statistical significance relative to wild-type was determined using two-way ANOVA and Tukey’s test. **** p < 0.0001. (B) Spore germination efficiency of the strains relative complemented with FLAG-tagged vs. wild-type complementation constructs. The number of colony forming units (CFUs) produced by germinating spores is shown. Average of results from three independent spore preparations are shown along with the associated standard deviations. Statistical analyses relative to the wild-type were performed using one-way ANOVA and Tukey’s test. ***, p < 0.001; **** p < 0.0001. (TIF) [file pgen.1009791.s005.tif]

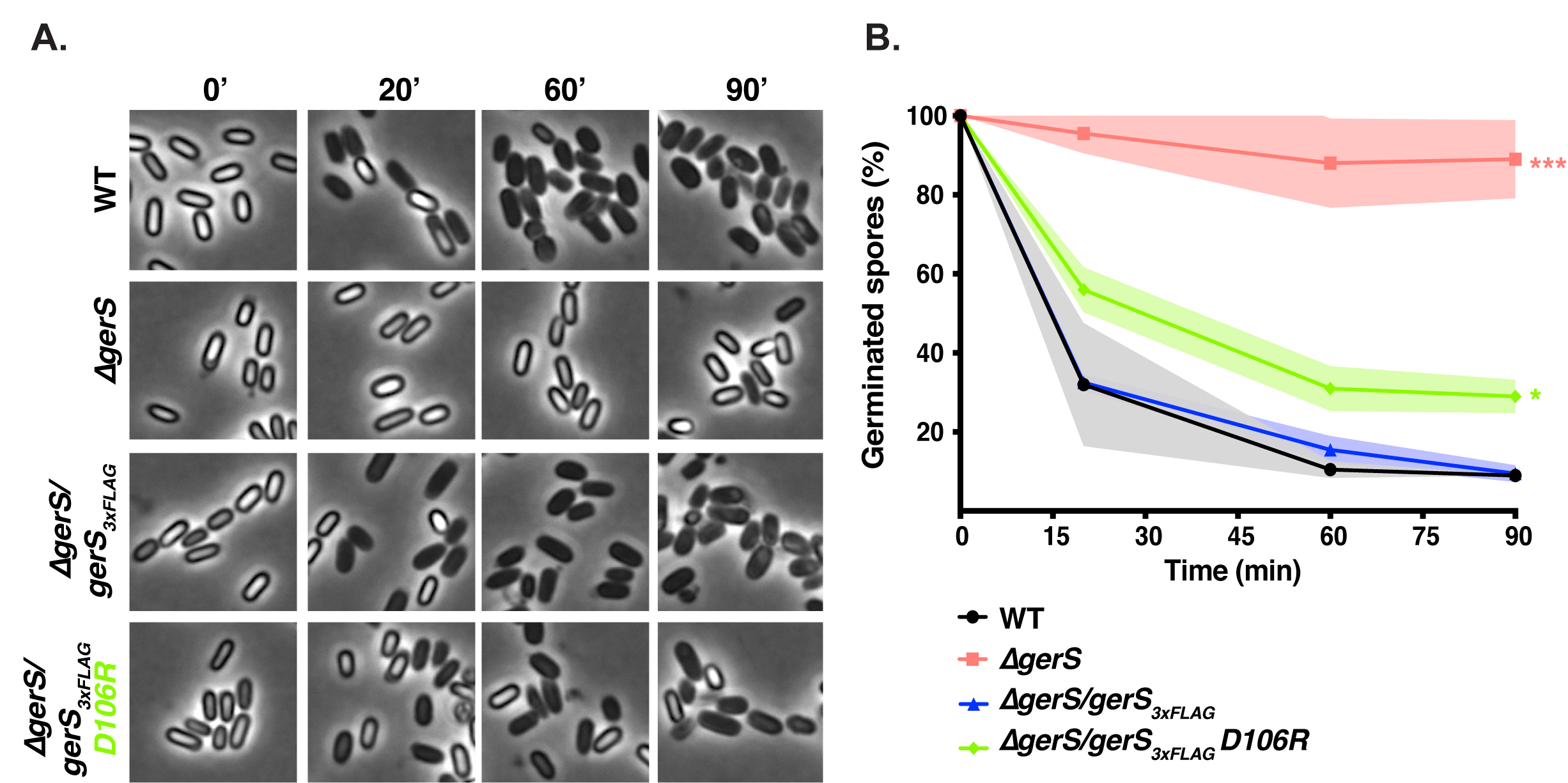

Supplement: S6 Fig — (A) Purified spores were resuspended in BHIS ± germinant and incubated aerobically for 90 min at 37°C. At the indicated time points samples were fixed in paraformaldehyde and visualized using phase-contrast microscopy. Scale bar, 1 um. B) Percent spore germination over time as detected by phase-contrast microscopy. The percentage of phase-bright spores of each strain at a given time point relative to the percentage at time zero is plotted. The mean of three assays from 2 independent spore preparations are shown. Shading represents the standard deviation as the area between error bars for each time point measured. Statistical significance relative to wild-type was determined using two-way ANOVA and Tukey’s test. **** p < 0.0001. (TIF) [file pgen.1009791.s006.tif]
