# Supplementary material for: A lipoprotein allosterically activates the CwlD amidase during Clostridioides difficile spore formation
Source: PLoS Genet. 2021 Sep 27;17(9):e1009791. doi: 10.1371/journal.pgen.1009791 (PMC8496864; doi:10.1371/journal.pgen.1009791)
Supplement: S1 Text — (DOCX) [file pgen.1009791.s007.docx]

**Supplementary Text S1 – *E. coli* strain construction**

**pET22b-*cwlD*_∆25_-H49A*-*His_6._** Primer pair #2545 and 2763 and primer pair #2762 and 2546 were used to amplify regions spanning the *cwlD* gene lacking its stop codon and first 25 codons. Primers 2763 and 2762 encode the H49A mutation. The PCR products were cloned into pET22b digested with NdeI/SalI using Gibson assembly. The Gibson was transformed into DH5α, and the resulting construct was sequenced verified before transforming the plasmid into BL21(DE3) for protein expression.

**pET22b-*cwlD*_∆25_-E65A*-*His_6._** Primer pair #2545 and 3309 and primer pair #3310 and 2546 were used to amplify regions spanning the *cwlD* gene lacking its stop codon and first 25 codons. Primers 3309 and 3310 encode the E65A mutation. The PCR products were cloned into pET22b digested with NdeI/SalI using Gibson assembly. The Gibson was transformed into DH5α, and the resulting construct was sequenced verified before transforming the plasmid into BL21(DE3) for protein expression.

**pET22b-*cwlD*_∆25_-E199A*-*His_6._** Primer pair #2545 and 3251 and primer pair #3252 and 2546 were used to amplify regions spanning the *cwlD* gene lacking its stop codon and first 25 codons. Primers 3251 and 3252 encode the E199A mutation. The PCR products were cloned into pET22b digested with NdeI/SalI using Gibson assembly. The Gibson was transformed into DH5α, and the resulting construct was sequenced verified before transforming the plasmid into BL21(DE3) for protein expression.

**pET22b-*cwlD*_∆25_-R169D*-*His_6._** Primer pair #2545 and 3731 and primer pair #3732 and 2546 were used to amplify regions spanning the *cwlD* gene lacking its stop codon and first 25 codons. Primers 3731 and 3732 encode the R169D mutation. The PCR products were cloned into pET22b digested with NdeI/SalI using Gibson assembly. The Gibson was transformed into DH5α, and the resulting construct was sequenced verified before transforming the plasmid into BL21(DE3) for protein expression.

**pET22b-*cwlD*_∆25_-R169Q*-*His_6._** Primer pair #2545 and 3727 and primer pair #3728 and 2546 were used to amplify regions spanning the *cwlD* gene lacking its stop codon and first 25 codons. Primers 3727 and 3728 encode the R169Q mutation. The PCR products were cloned into pET22b digested with NdeI/SalI using Gibson assembly. The Gibson was transformed into DH5α, and the resulting construct was sequenced verified before transforming the plasmid into BL21(DE3) for protein expression.

**pET22b-*cwlD*_∆25_-E78Q*-*His_6._** Primer pair #2545 and 3733 and primer pair #3734 and 2546 were used to amplify regions spanning the *cwlD* gene lacking its stop codon and first 25 codons. Primers 3733 and 3734 encode the E78Q mutation. The PCR products were cloned into pET22b digested with NdeI/SalI using Gibson assembly. The Gibson was transformed into DH5α, and the resulting construct was sequenced verified before transforming the plasmid into BL21(DE3) for protein expression.

**pET22b-*gerS*_∆22_-His_6._** Primer pair #3103 and 3104 was used to amplify the *gerS* gene lacking its stop codon and first 22 codons. The PCR product was cloned into pET22b digested with NdeI/SalI using Gibson assembly. The Gibson was transformed into DH5α, and the resulting construct was sequenced verified before transforming the plasmid into BL21(DE3) for protein expression.

**pET29a-*gerS*_∆22_-H61A.** Primer pair #3419 and 3725 and primer pair #3726 and 3420 were used to amplify regions spanning the *gerS* gene lacking its first 22 codons. Primers 3725 and 3726 encode the H61A mutation. The PCR products were cloned into pET29a digested with NdeI/XhoI using Gibson assembly. The Gibson was transformed into DH5α, and the resulting construct was sequenced verified before transforming the plasmid into BL21(DE3) for protein expression.

**pET29a-*gerS*_∆22_-D106R.** Primer pair #3419 and 3723 and primer pair #3724 and 3420 were used to amplify regions spanning the *gerS* gene lacking its first 22 codons. Primers 3723 and 3724 encode the D106R mutation. The PCR products were cloned into pET29a digested with NdeI/XhoI using Gibson assembly. The Gibson was transformed into DH5α, and the resulting construct was sequenced verified before transforming the plasmid into BL21(DE3) for protein expression.

**pET29a-*gerS*_∆22_-D106N.** Primer pair #3419 and 3719 and primer pair #3720 and 3420 were used to amplify regions spanning the *gerS* gene lacking its first 22 codons. Primers 3719 and 3720 encode the D106N mutation. The PCR products were cloned into pET29a digested with NdeI/XhoI using Gibson assembly. The Gibson was transformed into DH5α, and the resulting construct was sequenced verified before transforming the plasmid into BL21(DE3) for protein expression.

**pMTL-YN1C-*cwlD*_H49A._** Primer pair #2362 and 2763 and primer pair #2762 and 2450 were used to amplify regions spanning 274 bp upstream of *cwlD* and the *cwlD* gene including the stop codon.

Primers 2763 and 2762 encode the H49A mutation. The PCR products were cloned into pMTL-YN1C digested with NotI/XhoI using Gibson assembly.

**pMTL-YN1C-*cwlD*_E65A._** Primer pair #2362 and 3309 and primer pair #3310 and 2450 were used to amplify regions spanning 274 bp upstream of *cwlD* and the *cwlD* gene including the stop codon.

Primers 3309 and 3310 encode the E65A mutation. The PCR products were cloned into pMTL-YN1C digested with NotI/XhoI using Gibson assembly.

**pMTL-YN1C-*cwlD*_E199A._** Primer pair #2362 and 3251 and primer pair #3252 and 2450 were used to amplify regions spanning 274 bp upstream of *cwlD* and the *cwlD* gene including the stop codon.

Primers 3251 and 3252 encode the E199A mutation. The PCR products were cloned into pMTL-YN1C digested with NotI/XhoI using Gibson assembly.

**pMTL-YN1C-*cwlD*-3xFLAG_._** To clone the *cwlD* complementation construct encoding a C-terminal FLAG_3_ epitope tag, primer pair #2362 and 2599 was used to amplify the *cwlD* gene without the stop codon and its promoter region (274 bp upstream) along with sequence encoding part of a FLAG epitope. The resulting PCR product was assembled with the following g-block encoding the FLAG_3_ epitope into pMTL-YN1C along with digested with NotI and XhoI using Gibson assembly.

gBlock: Cdif CwlD-3xFLAG for pMTL-YN1C

CAGAGAAGTCAAACCAAGGGATGATATATATCTTTTGAAAGACAATAATATTCCATCAGTACTGATAGAATGTGGTTTTTTATCAAATGAAAAAGAGTGTAAACTCTTAACTGATGAAACATATCAAGAAAAAATAGCATGGGCAATCTACATAGGAATACAAAAATATTTAAGTGATTATAAAGATGATGATGATAAAGACTATAAAGATGACGATGATAAGGATTATAAGGATGATGATGACAAATAACTCGAGGCCTGCAGACATGCAAGC

**pMTL-YN1C-*cwlD*_H49A_-3xFLAG_._** Primer pair #2362 and 2763 and primer pair #2762 and 2562 were used to amplify the *cwlD* gene without the stop codon and its promoter region (274 bp upstream) along with sequence encoding the FLAG_3_ epitope using the pMTL-YN1C-*cwlD*-3xFLAG as template. Primers 2763 and 2762 encode the H49A mutation. The PCR products were cloned into pMTL-YN1C digested with NotI/XhoI using Gibson assembly.

**pMTL-YN1C-*cwlD*_E199A_-3xFLAG_._** Primer pair #2362 and 3251 and primer pair #3252 and 2562 were used to amplify the *cwlD* gene without the stop codon and its promoter region (274 bp upstream) along with sequence encoding the FLAG_3_ epitope using the pMTL-YN1C-*cwlD*-3xFLAG as template. Primers 3251 and 3252 encode the E199A mutation. The PCR products were cloned into pMTL-YN1C digested with NotI/XhoI using Gibson assembly.

**pMTL-YN1C-*cwlD*_R169D_-3xFLAG_._** Primer pair #2362 and 3731 and primer pair #3732 and 2562 were used to amplify the *cwlD* gene without the stop codon and its promoter region (274 bp upstream) along with sequence encoding the FLAG_3_ epitope using the pMTL-YN1C-*cwlD*-3xFLAG as template. Primers 3731 and 3732 encode the R169D mutation. The PCR products were cloned into pMTL-YN1C digested with NotI/XhoI using Gibson assembly.

**pMTL-YN1C-*gerS*-3xFLAG-*alr2*_._** To clone the *gerS* complementation construct encoding a C-terminal FLAG_3_ epitope tag, primer pair #2181 and 2365 was used to amplify the *gerS* gene without the stop codon and its promoter region (367 bp upstream) along with sequence encoding part of a FLAG epitope. The resulting PCR product was assembled with the following g-block encoding the FLAG_3_ epitope. The *alr2* gene was amplified using primer pair #2617 and 2408. The two PCR fragments were cloned into pMTL-YN1C digested with NotI and XhoI using Gibson assembly.

gBlock: Cdif GerS-3xFLAG-alr2 for pMTL-YN1C

GAGGAGATGAAAGTAAAGTTATCCAAAGGACATTTGGTTTTAGAGACTTTTATTCCTGGGGATAATAAATATTTTAACAAGCAAGTATTATATGTAAATGCTGACACAAAAAATCCTGAAAAAATGGAAGTGTTAGATAAAGAGGGAGTGCCAAGATTTACAGTAAAATACAAAGATTTTGAATACAGAAACGATTATAAGGATGACGATGATAAAGACTATAAAGATGACGATGATAAGGATTATAAGGATGACGATGACAAATAACTCGAGGCCTGCAGACATGCAAGCTTGGC

**pMTL-YN1C-*gerS_D106R_*-3xFLAG-*alr2*_._** Primer pair #2181 and 3723 and primer pair #3724 and 2408 were used to amplify the *gerS* gene without the stop codon and its promoter region (367 bp upstream) along with sequence encoding the FLAG_3_ epitope and the *alr2* gene using the pMTL-YN1C-*cwlD*-3xFLAG-*alr2* as template. Primers 3723 and 3724 encode the D106R mutation. The PCR products were cloned into pMTL-YN1C digested with NotI/XhoI using Gibson assembly.
