# Supplementary material for: A lipoprotein allosterically activates the CwlD amidase during Clostridioides difficile spore formation
Source: PLoS Genet. 2021 Sep 27;17(9):e1009791. doi: 10.1371/journal.pgen.1009791 (PMC8496864; doi:10.1371/journal.pgen.1009791)
Supplement: S2 Table — (DOCX) [file pgen.1009791.s010.docx]

**S2 Table. Strains and plasmids used in this study.**

| **Strain #** | **Strain name** | **Relevant genotype or features** | **Source/ reference** |
| --- | --- | --- | --- |
| ***C. difficile* strains – 630∆erm** | | | |
| 756 | 630∆*erm*∆*pyrE* | *erm*-sensitive derivate of 630 with a deletion in *pyrE* | (1) |
| 846 | 630∆*erm*-p | *erm*-sensitive derivate of 630 with *pyrE* restored | (2) |
| 849 | 630∆*erm ∆spo0A*-p | 630∆*erm* ∆*spo0A* with *pyrE* restored | (2) |
| 950 | 630∆*erm* ∆*gerS*∆*pyrE* | 630∆*erm*∆*pyrE* with *gerS* deleted (*CD630_34640*) | (3) |
| 1075 | 630∆*erm* ∆*gerS*-p | 630∆*erm* ∆*gerS* with *pyrE* restored | (3) |
| 1617 | 630∆*erm* ∆*cwlD∆pyrE* | 630∆*erm*∆*pyrE* with *cwlD* deleted (*CD630_01060*) | (3) |
| 1677 | 630∆*erm* ∆*gerS*/*gerS-alr2* | 630∆*erm* ∆*gerS* with *gerS-alr2* in the *pyrE* locus | (3) |
| 1686 | 630∆*erm* ∆*cwlD*-p | 630∆*erm* ∆*cwlD* with *pyrE* restored | (3) |
| 1692 | 630∆*erm* ∆*cwlD*/*cwlD* | 630∆*erm* ∆*cwlD* with *cwlD* in the *pyrE* locus | (3) |
| 2053 | 630∆*erm- p-*∆gerS*/gerS-*3xFLAG*-alr2* | 630∆*erm* ∆gerS with gerS*-*3xFLAG-*alr2* in the *pyrE* locus | This study |
| 2056 | 630∆*erm* ∆*cwlD*/*cwlD-*3xFLAG | 630∆*erm* ∆*cwlD* with *cwlD-*3xFLAG in the *pyrE* locus | This study |
| 2161 | 630∆*erm* ∆*cwlD*/*cwlD_H49A_* | 630∆*erm* ∆*cwlD* with *cwlD_H49A_* in the *pyrE* locus | This study |
| 2764 | 630∆*erm* ∆*cwlD*/*cwlD_E199A_* | 630∆*erm* ∆*cwlD* with *cwlD_E199A_* in the *pyrE* locus | This study |
| 2900 | 630∆*erm* ∆*cwlD*/*cwlD_E65A_* | 630∆*erm* ∆*cwlD* with *cwlD_E65A_* in the *pyrE* locus | This study |
| 2918 | 630∆*erm* ∆*cwlD*/*cwlD_H49A_-*3xFLAG | 630∆*erm* ∆*cwlD* with *cwlD_H49A_-*3xFLAG in the *pyrE* locus | This study |
| 2924 | 630∆*erm* ∆*cwlD*/*cwlD_E199A_-*3xFLAG | 630∆*erm* ∆*cwlD* with *cwlD_E199A_-*3xFLAG in the *pyrE* locus | This study |
| 3549 | 630∆*erm* ∆*gerS/gerS_D106R_-*3xFLAG | 630∆*erm* ∆*cwlD* with *gerS_D106R_-*3xFLAG-*alr2* in the *pyrE* locus | This study |
| 3564 | 630∆*erm* ∆*cwlD*/*cwlD_R169D_-*3xFLAG | 630∆*erm* ∆*cwlD* with *cwlD_R169D_-*3xFLAG in the *pyrE* locus | This study |
| ***E. coli* strains** | | | |
| 41 | DH5a | F– Φ80*lacZ*ΔM15 Δ(*lacZYA-argF*) U169 *recA1 endA1 hsdR17* (rK–, mK+) *phoA supE44* λ– *thi-1 gyrA96 relA1* | D. Cameron |
| 531 | HB101/pRK24 | F- *mcrB mrr hsdS20*(rB–mB–) *recA13 leuB6 ara-13 proA2 lavYI galK2 xyl-6 mtl-1 rpsL20* carrying pRK24 | C. Ellermeier |
| 892 | BL21 (DE3) | F– *ompT hsdSB*(rB–mB–) *gal dcm* (DE3) | C. Ellermeier |
|  | B834 (DE3) | F^-^ *ompT hsdS*_B_(r_B_^-^ m_B_^-^) *gal dcm met* (DE3) | Novagen |
| 2045 | pET22b-∆25-*cwlD-*His_6_ | pET22b-*cwlD* (deletion of N-terminal 25 aa with His6 tag) in BL21(DE3) | (3) |
| 2147 | pMTL-YN1C-*gerS-*3xFLAG-*alr2* | pMTL-YN1C- *gerS-*3xFLAG in HB101/pRK24 | This study |
| 2149 | pMTL-YN1C-*cwlD-*3xFLAG | pMTL-YN1C-*cwlD-*3xFLAG in HB101/pRK24 | This study |
| 2191 | pMTL-YN1C-*cwlD_H49A_* | pMTL-YN1C-*cwlD_H49A_* in HB101/pRK24 | This study |
| 2219 | pET22b-∆25-*cwlD_H49A_-*His_6_ | pET22b-*cwlD* (deletion of N-terminal 25 aa and H49A point mutation with His6 tag) in BL21(DE3) | This study |
| 2395 | pET22b-∆22-*gerS*-His_6_ | pET22b-gerS (deletion of N-terminal 22 aa with His6 tag) in BL21(DE3) | This study |
| 2493 | pMTL-YN1C-*cwlD_E199A_* | pMTL-YN1C-*cwlD_E199A_* in HB101/pRK24 | This study |
| 2557 | pMTL-YN1C-*cwlD_E65A_* | pMTL-YN1C-*cwlD_E65A_* in HB101/pRK24 | This study |
| 2566 | pMTL-YN1C-*cwlD_H49A_-*3xFLAG | pMTL-YN1C-*cwlD_H49A_-*3xFLAG in HB101/pRK24 | This study |
| 2570 | pMTL-YN1C-*cwlD_E199A_-*3xFLAG | pMTL-YN1C-*cwlD_E199A_-*3xFLAG in HB101/pRK24 | This study |
| 2574 | pET22b-∆25-*cwlD_E65A_-*His_6_ | pET22b-*cwlD* (deletion of N-terminal 25 aa and E65A point mutation with His6 tag) in BL21(DE3) | This study |
| 2576 | pET22b-∆25-*cwlD_E199A_-*His_6_ | pET22b-*cwlD* (deletion of N-terminal 25 aa and E199A point mutation with His6 tag) in BL21(DE3) | This study |
| 2587 | pET22b-∆25-*cwlD-*His_6_ + pET29a ∆22-*gerS*+TAA | Co-expression of pET22b-*cwlD* (deletion of N-terminal 25 aa with His6 tag) with pET29a-*gerS* (untagged deletion of N-terminal 22 aa) in BL21(DE3) | (3) |
| 2588 | pET22b-∆25-*cwlD_H49A_-*His_6_ + pET29a ∆22-*gerS*+TAA | Co-expression of pET22b-*cwlD* (deletion of N-terminal 25 aa and H49A point mutation with His6 tag) with pET29a-*gerS* (untagged deletion of N-terminal 22 aa) in BL21(DE3) | This study |
| 2589 | pET22b-∆25-*cwlD_E65A_-*His_6_ + pET29a ∆22-*gerS*+TAA | Co-expression of pET22b-*cwlD* (deletion of N-terminal 25 aa and E65A point mutation with His6 tag) with pET29a-*gerS* (untagged deletion of N-terminal 22 aa) in BL21(DE3) | This study |
| 2590 | pET22b-∆25-*cwlD_E199A_-*His_6_ + pET29a ∆22-*gerS*+TAA | Co-expression of pET22b-*cwlD* (deletion of N-terminal 25 aa and E199A point mutation with His6 tag) with pET29a-*gerS* (untagged deletion of N-terminal 22 aa) in BL21(DE3) | This study |
| 2593 | pET22b-∆25-*cwlD-*His_6_ + pET29a ∆22-*gerS*+TAA | Co-expression of pET22b-*cwlD* (deletion of N-terminal 25 aa with His6 tag) with pET29a-*gerS* (untagged deletion of N-terminal 22 aa) in B834 (DE3) | This study |
| 2984 | pET22b-∆25-*cwlD_E78Q_-*His_6_ + pET29a ∆22-*gerS*+TAA | Co-expression of pET22b-*cwlD* (deletion of N-terminal 25 aa and E78Q point mutation with His6 tag) with pET29a-*gerS* (untagged deletion of N-terminal 22 aa) in BL21(DE3) | This study |
| 2987 | pET22b-∆25-*cwlD_R169D_-*His_6_ + pET29a ∆22-*gerS*+TAA | Co-expression of pET22b-*cwlD* (deletion of N-terminal 25 aa and R169D point mutation with His6 tag) with pET29a-*gerS* (untagged deletion of N-terminal 22 aa) in BL21(DE3) | This study |
| 2993 | pET22b-∆25-*cwlD-*His_6_ + pET29a ∆22-*gerS_D106R_*+TAA | Co-expression of pET22b-*cwlD* (deletion of N-terminal 25 aa with His6 tag) with pET29a-*gerS* (untagged deletion of N-terminal 22 aa with D106R point mutation) in BL21(DE3) | This study |
| 2994 | pET22b-∆25-*cwlD-*His_6_ + pET29a ∆22-*gerS_H61A_*+TAA | Co-expression of pET22b-*cwlD* (deletion of N-terminal 25 aa with His6 tag) with pET29a-*gerS* (untagged deletion of N-terminal 22 aa with H61A point mutation) in BL21(DE3) | This study |
| 2996 | pET22b-∆25-*cwlD_R169D_-*His_6_ + pET29a ∆22-*gerS_D106R_*+TAA | Co-expression of pET22b-*cwlD* (deletion of N-terminal 25 aa and R169D point mutation with His6 tag) with pET29a-*gerS* (untagged deletion of N-terminal 22 aa with D106R point mutation) in BL21(DE3) | This study |
| 3008 | pMTL-YN1C-*cwlD_R169Q_-*3xFLAG | pMTL-YN1C-*cwlD_R169Q_-*3xFLAG in HB101/pRK24 | This study |
| 3009 | pMTL-YN1C-*cwlD_R169D_-*3xFLAG | pMTL-YN1C-*cwlD_R169D_-*3xFLAG in HB101/pRK24 | This study |
| 3010 | pMTL-YN1C-*gerS_D106N_-*3xFLAG-*alr2* | pMTL-YN1C-*gerS_D106N_-*3xFLAG-*alr2* in HB101/pRK24 | This study |
| 3011 | pMTL-YN1C-*gerS_D106R_-*3xFLAG-*alr2* | pMTL-YN1C-*gerS_D106R_-*3xFLAG-*alr2* in HB101/pRK24 | This study |

**Plasmids**

| pET22b | For cloning His-tagged expression constructs | Novagen |
| --- | --- | --- |
| pET29a | For cloning His-tagged expression constructs | Novagen |
| pMTL-YN1C | For cloning complementation constructs to be integrated into the pyrE locus of 630∆*erm*∆*pyrE* | (1) |

**References**

1. Ng YK, Ehsaan M, Philip S, Collery MM, Janoir C, Collignon A, Cartman ST, Minton NP. 2013. Expanding the repertoire of gene tools for precise manipulation of the *Clostridium difficile* genome: allelic exchange using *pyrE* alleles. PLoS One 8:e56051.

2. Donnelly ML, Li W, Li YQ, Hinkel L, Setlow P, Shen A. 2017. A *Clostridium difficile*-Specific, Gel-Forming Protein Required for Optimal Spore Germination. mBio 8.

3. Diaz OR, Sayer CV, Popham DL, Shen A. 2018. *Clostridium difficile* Lipoprotein GerS Is Required for Cortex Modification and Thus Spore Germination. mSphere 3.
