# Supplementary material for: A lipoprotein allosterically activates the CwlD amidase during Clostridioides difficile spore formation
Source: PLoS Genet. 2021 Sep 27;17(9):e1009791. doi: 10.1371/journal.pgen.1009791 (PMC8496864; doi:10.1371/journal.pgen.1009791)
Supplement: S3 Table — (DOCX) [file pgen.1009791.s011.docx]

**S3 Table: Primers used in this study.**

| **Number** | **Primer Name** | **Primer Sequence** |
| --- | --- | --- |
| 2181 | 5' NotI *gerS* Gibson | GGAATTAGGGATGTAATAA**GCGGCCGC**CAGTTGTAGATTCAGAGAATAGAGTTG |
| 2362 | 5' NotI P*_cwlD_* Gibson | GGAATTAGGGATGTAATAA**GCGGCCGC**ATAAGGTTATATATTTTAAGATATTATTTAAC |
| 2365 | 3' gerS rev eos | CTCTAAAACCAAATGTCCTTTGGATAACTTTACTTTCATCTCCTC |
| 2408 | 3' XhoI *gerS* downstream Gibson | CAAGCTTGCATGTCTGCAGGC**CTCGAG**TTATTTTAGCAAATAACTGTTTATTTG |
| 2450 | 3' XhoI *cwlD* YN1C | CAAGCTTGCATGTCTGCAGGC**CTCGAG**TCAACTTAAATATTTTTGTATTCCTATGTAG |
| 2545 | 5' Δ25aa-*cwlD* Gibson NdeI pET22b | GTTTAACTTTAAGAAGGAGATATA**CATATG**AAAAATATTTCTGAAGATGTTATCAAG |
| 2546 | 3' Δ25aa-*cwlD* Gibson SalI pET22b | GCTCGAGTGCGGCCGCAAGCTT**GTCGAC**ACTTAAATATTTTTGTATTCCTATGTAGATTG |
| 2562 | 3' XhoI FLAG YN1C | CAAGCTTGCATGTCTGCAGGC**CTCGAG**TTATTTGTCATCATCATCCTTATAATCC |
| 2599 | 3' *cwlD*-3XFLAG rev eos | CATCCCTTGGTTTGACTTCTCTGTTGTTTGTCTTATCTACTACTCTTTTAAGTTCTTC |
| 2617 | 5' *gerS*-3xFLAG-alr2 Gibson SOE | GGATTATAAGGATGACGATGACAAATAAGGGGGACTAAAGACATGCAAAAAATAACAGTG |
| 2762 | 5' *cwlD* (H49A) SOE | CAAAACTATAATTTTAGATGCTGGTGCAGGAGGCATTGATCCAGGTGCATTAAATAAG |
| 2763 | 3' *cwlD* (H49A) eos | CTTATTTAATGCACCTGGATCAATGCCTCCTGCACCAGCATCTAAAATTATAGTTTTG |
| 3103 | ∆22-*gerS* Gibson NdeI pET22 | GTTTAACTTTAAGAAGGAGATATA**CATATG**CAAAAACGACAGTCCACAAAAGAAGAAG |
| 3104 | ∆22-*gerS* (no stop) Gibson SalI pet22b rev | CTCGAGTGCGGCCGCAAGCTT**GTCGAC**GTTTCTGTATTCAAAATCTTTGTATTTTAC |
| 3251 | 3' *cwlD* E199A eos | CACTCTTTTTCATTTGATAAAAAACCACATGCTATCAGTACTGATGGAATATTATT |
| 3252 | 5' c*wlD* E199A SOE | AATAATATTCCATCAGTACTGATAGCATGTGGTTTTTTATCAAATGAAAAAGAGTG |
| 3309 | 3' *cwlD* E65A eos | TAAGTGTTATTGCTAGGTTAATATCCTTTGCAGATGTACTCTTATCCTTATTTAATG |
| 3310 | 5' *cwlD* E65A SOE | CATTAAATAAGGATAAGAGTACATCTGCAAAGGATATTAACCTAGCAATAACACTTA |
| 3419 | 5’ ∆22aa-*gerS* NdeI pet29 | GTTTAACTTTAAGAAGGAGATATA**CATATG**AGAAAAAAGTGGACCATAGTATGTAT |
| 3420 | 3’ ∆22aa-*gerS* XhoI pet29 | GATCTCAGTGGTGGTGGTGGTGGTG**CTCGAG**TCAGTTTCTGTATTCAAAATCTTTGTAT |
| 3719 | 3' gerS D106N | ATTTTTTCCTGTATTAGGTAACTCCACTACATTACTAATCTTTGGATTTTTAACTAATA |
| 3720 | 5' gerS D106N | TATTAGTTAAAAATCCAAAGATTAGTAATGTAGTGGAGTTACCTAATACAGGAAAAAAT |
| 3723 | 3' *gerS* D160R eos | TTTTTTCCTGTATTAGGTAACTCCACTACACGACTAATCTTTGGATTTTTAACTAATA |
| 3724 | 5' gerS D160R SOE | TATTAGTTAAAAATCCAAAGATTAGTCGTGTAGTGGAGTTACCTAATACAGGAAAAAA |
| 3725 | 3' *gerS* H61A eos | TTTTTTATAAGTATGAATTAAAACATAATTAGCTGGGCTTTTATTTCCAACTACTTCTA |
| 3726 | 5' *gerS* H61A SOE | TAGAAGTAGTTGGAAATAAAAGCCCAGCTAATTATGTTTTAATTCATACTTATAAAAAA |
| 3727 | 3' *cwlD* R169Q eos | GACTTCTCTGTTGTTTGTCTTATCTACTACTTGTTTAAGTTCTTCTTGAATACACTTCG |
| 3728 | 5' *cwlD* R169Q SOE | CGAAGTGTATTCAAGAAGAACTTAAACAAGTAGTAGATAAGACAAACAACAGAGAAGTC |
| 3731 | 3' *cwlD* R169D eos | GACTTCTCTGTTGTTTGTCTTATCTACTACATCTTTAAGTTCTTCTTGAATACACTTCG |
| 3732 | 5' *cwlD* R169D SOE | CGAAGTGTATTCAAGAAGAACTTAAAGATGTAGTAGATAAGACAAACAACAGAGAAGTC |
| 3733 | 3' *cwlD* E78Q eos | TATTACAAGGCCACCACTTGATTCTATAAGTTGTCTAAGCTTAAGTGTTATTGCTAGGT |
| 3734 | 5' *cwlD* E78Q SOE | ACCTAGCAATAACACTTAAGCTTAGACAACTTATAGAATCAAGTGGTGGCCTTGTAATA |

Restriction sites are underlined and in bold.
