# Supplementary material for: A lipoprotein allosterically activates the CwlD amidase during Clostridioides difficile spore formation
Source: PLoS Genet. 2021 Sep 27;17(9):e1009791. doi: 10.1371/journal.pgen.1009791 (PMC8496864; doi:10.1371/journal.pgen.1009791)
Supplement: S4 Table — (DOCX) [file pgen.1009791.s012.docx]

**S4 Table: Crystallographic data collection and structure refinement statistics for CwlD:GerS.**

|  | Native (*L8*) | SeMet (*R9*) | Iod (*R13*) |
| --- | --- | --- | --- |
| PDB ID |  | *ref113* |  |
| Data Processing Proteum |  |  |  |
| Wavelength | 1.033 | 0.9794 | 0.9794 |
| Space Group  Cell a=b, c (Å) | P6(5)22  104.6, 156.2 | P6(5)22  105.2, 154.8 | P6(5)22  10.65, 156.5 |
| Resolution (Å)  (High resolution)^a^ | 45 - 2.40  (2.48 - 2.40) | 45 - 2.40  (2.49-2.40) | 46 - 2.49  (2.58 - 2.59) |
| Reflections | 20346 | 20401 | 18805 |
| Completeness (%) | 99.5 (98.4) | 99.9 (99.2) | 99.5 (95.3) |
| Wilson B | 72.9 | 59.9 | 69.8 |
| Multiplicity | 19.0 (19,.4) | 18.7 (18.3) | 19.0 (19.6) |
| I/sigI | 14.1 (1.0) | 14.5 (1.5) | 14.4 (1.5) |
| R_meas_ % | 15.1 (261) | 12.4 (231) | 10.3 (256) |
| R_pim_ % | 3.5 (59.,1) | 2.9 (53.6) | 2.4 (57.6) |
| CC^1/2^ | 0.997 (0.345) | 0.999 (0.572) | 0.999 (0.644) |
| Phasing |  |  |  |
| Heavy Atom Sites Sharp45  CC_all_/CC_weak_  CFOM  Correlation |  | 6  56.8/34.8  91.6  0.501 | 12  41.5/9.4  50.9  0.363 |
| Phasing Power  Isomorphous  Anomalous |  | 1.02  2.73 | 0.64  0.64 |
| Phasing R-cullis acentric  Isomorphous  Anomalous |  | 0.275  0.464 | 0.621  0.893 |
| Density Modification FOM |  | 0.63 |  |
| Model refinement |  |  |  |
| R_work_/R_free_ % |  | 20.3/24.9 |  |
| RMS Bonds (Å)  RMS Angles (^o^) |  | 0.008  1.00 |  |
| Ramachandran (%)  Favored  Outlier |  | 95.8  0.3 |  |
| Mean B (Å^2^) |  | 69.2 |  |
| Correlation Coefficient |  | 0.823 |  |
| Atoms Protein  Ligand (Zn^2+^)  Solvent |  | 2890  1  18 |  |
| Coordinate error (Å)  Phase error (^o^) |  | 0.39  30.1 |  |

^a^Numbers in parentheses denote high resolution bin.
