# Supplementary material for: A lipoprotein allosterically activates the CwlD amidase during Clostridioides difficile spore formation
Source: PLoS Genet. 2021 Sep 27;17(9):e1009791. doi: 10.1371/journal.pgen.1009791 (PMC8496864; doi:10.1371/journal.pgen.1009791)
Supplement: Attachment — Submitted filename: 2021-08-17 Response to Reviews.docx [file pgen.1009791.s013.docx]

**Response to Reviewer Comments**

*We greatly appreciate that Reviewers thoughtful and careful consideration of the manuscript. We have made the requested changes and believe the revised manuscript is improved in its readability and consideration of alternative interpretations of the data.*

**Reviewer #1**: The manuscript by Feliciano et al. reports the first co-crystal structure of the soluble portions of a cell wall amidase (CwlD) with its cognate regulator (GerS). These proteins are involved in maturation of the spore peptidoglycan in C. difficile. The structure revealed that the GerS/CwlD complex displays a 2:2 stoichiometry and that GerS does not bindi near the active site of CwlD. Instead, in vitro experiments suggested that GerS regulates CwlD activity by modulating the ability of CwlD to bind Zn2+, and that disrupting Zn2+ binding via mutagenesis disrupts complex assembly. The authors demonstrated that CwlD variants which were incapable of binding Zn2+ exhibited a germination defect in vivo, likely due to the reduced protein stability of CwlD and GerS. Finally, guided by the structure, the authors identified two salt bridges that were critical for GerS binding to CwlD, and report that disrupting one of them retarded the speed of germination. The paper was well-written in a way that should be accessible to a general audience, and I imagine that the results will be of broad interest to bacterial cell and developmental biologists, enzymologists, and those who study bacterial pathogenesis. I have very few suggestions for improvement that the authors may or may not choose to incorporate.

**Major comment:
1. Lines 249-251, Fig. 3E**. The authors conclude that disrupting the Zn2+ binding site does not disrupt the stoichiometry of the complex (based on Coomassie staining in Fig. 3E), but to my eye it appears that the amount of CwlD in peak 1 is reduced relative to WT, especially with the H49A and the E65A variants. I realize the change in MW of the peak is annoyingly ~half of the MW of a single CwlD molecule, but perhaps the authors may consider raising the possibility that stoichiometry is affected, in addition to their preferred model that there is a conformational change upon disrupting Zn2+ binding? (optional)

- *We have added the following text to the lines highlighted: “…*although we cannot rule out the possibility that the CwlD Zn^2+^ binding mutations affect the stoichiometry of CwlD binding to GerS.*.*

Minor comments:
**1. Figure 1**. Please define in the figure legend the “25” next to the Coomassie-stained gels (presumably molecular weight marker?).

- *The figure legend now indicates that “25” is the molecular weight marker.*

2. The bound divalent cation is referenced at different places in the paper as “zinc”, “Zn”, or “Zn2+”. **For consistency, perhaps simply stick to “Zn2+”** or “Zn2+ ion”?

- *We have changed the designation of the divalent cation so that it is only referrred to as “Zn^2+^*” in the manuscript.

**Reviewer #2**: This manuscript is an extremely well written, very clearly laid out, and comprehensive analysis of the genetics, biochemistry, and structure of the CwlD:GerS complex of Clostridium difficile. The authors show that GerS binds to CwlD and that the interaction allows conjugation of CwlD to a critical Zn active site cofactor. In turn, Zn binding preserves the CwlD:GerS interaction and stabilizes the GerS protein in vivo. They also establish an important contact salt bridge and even show that charge reversal on each protein partially reconnects the interaction. All told the work is a rather complete example of how peptidoglycan modification/hydrolase activity (CwlD) is allosterically regulated by another factor as well as how that interaction governs biochemistry and ultimately physiological role in spores. The data are beautiful. **The only complaint I have is that the paper feels a tad long**.

- *We thank the Reviewer for this critique. We have shortened the Discussion section, which was a little repetitive, by ~350 words. We believe the changes improve the manuscript’s readability.*

**Line 125**. One thing that I didn’t quite understand in the paper starts here. This line seems to suggest a 2:1 ratio of GerS:CwlD in SEC but later experiments DLS and 3D structure support a 2:2 ratio. Also, I didn’t understand how the “vice versa” would also satisfy the size of the complex. I don’t think this is a major issue, I just didn’t understand why the results were different or how it was being reconciled.

- *We have replaced “vice versa” with “…a CwlD dimer and GerS monomer” and corrected the MW for the GerS dimer to be 46 kDa (instead of 45 kDa) and the CwlD monomer as 28 kDa (instead of apparent MW of 33 kDa originally listed) and detected by size exclusion chromatography.* *We recognize that the Reviewer’s confusion understandably arose from the difference in MW size listed – in the text we had listed the apparent MW based on the MW standard curve we had measured for our column rather than the predicted MW.*

**Line 160**. I don’t think DLS has been defined at this point in the manuscript. Move acronym definition forward.

- *We now indicate that “DLS” is an abbreviation for “dynamic light scattering”.*

It is brought up a few times in the manuscript that homologs in *Bacillus* etc. do not have a cognate GerS and do not require activation. That doesn’t preclude the possibility that GerS-lacking organisms do not yet encode regulators of CwlD, an inhibitor for example.

- *The Reviewer raises an excellent point. We have added the following statement to the Discussion section: lines 588-589 “it is also possible that B. subtilis (or other organisms lacking GerS homologs) encode an unknown regulator that modulates CwlD activity.”*
